# Apigenin Protects Against Cisplatin-Induced Cardiotoxicity: Potential Involvement of CD38-Sirt3 Signaling in Rats

**DOI:** 10.3390/molecules31132300

**Published:** 2026-07-01

**Authors:** Natticha Sumneang, Jannarong Intakhad, Worakan Boonhoh, Arnon Pudgerd, Orawan Wongmekiat, Anongporn Kobroob

**Affiliations:** 1 Department of Medical Science, School of Medicine, Walailak University, Nakhon Si Thammarat 80160, Thailand; natticha.su@wu.ac.th; 2 Center of Excellence in Tropical Pathobiology, Walailak University, Nakhon Si Thammarat 80160, Thailand; 3 Faculty of Medicine, Vongchavalitkul University, Nakhon Ratchasima 30000, Thailand; jannarong_int@vu.ac.th; 4 Akkhraratchakumari Veterinary College, Walailak University, Nakhon Si Thammarat 80160, Thailand; worakan.bo@wu.ac.th; 5 Division of Anatomy, School of Medical Sciences, University of Phayao, Phayao 56000, Thailand; arnon.pu@up.ac.th; 6 Integrative Renal Research Unit, Department of Physiology, Faculty of Medicine, Chiang Mai University, Chiang Mai 50200, Thailand; orawan.wongmekiat@cmu.ac.th; 7 Division of Physiology, School of Medical Sciences, University of Phayao, Phayao 56000, Thailand

**Keywords:** heart, cisplatin, apigenin, flavonoids, CD38, Sirt3, oxidative stress, inflammation, apoptosis

## Abstract

Background: Cisplatin-induced cardiotoxicity is associated with oxidative stress, inflammation, and apoptosis; however, the role of CD38-Sirt3 signaling remains unclear. This study investigated whether apigenin protects against cisplatin-induced cardiac injury via modulation of CD38-Sirt3 signaling. Methods: Male Sprague Dawley rats were assigned to three groups, (1) Control, (2) Cisplatin (5 mg/kg), and (3) Pretreatment with apigenin (50 mg/kg/day) plus cisplatin groups. Then, left ventricular (LV) function, cardiac injury, oxidative stress, inflammation, apoptosis, and CD38-Sirt3 signaling-related proteins were assessed. Results: Cisplatin impaired LV function and induced cardiac injury, oxidative stress, inflammation, and apoptosis in rats. These changes were accompanied by increased cardiac CD38 and decreased cardiac Sirt3 and SOD2 expression. Apigenin significantly improved LV function (%LVEF and %LVFS), reduced cardiac injury (LDH, CK-MB), attenuated oxidative stress, suppressed inflammatory responses (TNF-α, IL-1β, p-NF-κB, TLR-4), and inhibited apoptosis (Bax/Bcl-2, cleaved caspase-3). Notably, apigenin improved cardiac SOD2 expression and reversed the alteration of CD38-Sirt3 signaling in cisplatin-treated rats. Conclusions: This study provides evidence that cisplatin-induced cardiotoxicity is associated with alterations in CD38-Sirt3 signaling. Apigenin attenuated LV dysfunction and cardiac injury, reduced oxidative stress, inflammation, and apoptosis, potentially through CD38-Sirt3 signaling. These findings highlight the cardioprotective potential of apigenin against cisplatin-induced cardiotoxicity.

## 1. Introduction

Cisplatin is one of the most widely used chemotherapeutic agents for the treatment of various types of cancers, such as lung, colon, ovarian, and testicular cancers [1,2]. It exerts anticancer effects by damaging deoxyribonucleic acid (DNA) and inhibiting DNA synthesis, leading to cancer cell death [3,4]. Although cisplatin exhibits potent anticancer efficacy, its clinical use is limited by dose-dependent systemic toxicity [3,5]. Among its adverse effects, cardiotoxicity has emerged as a major concern, characterized by myocardial injury, left ventricular (LV) dysfunction, and cardiac electrical abnormalities [5,6,7,8,9,10].

Evidence suggests that oxidative stress plays a major role in cisplatin-induced cardiotoxicity [1,2]. Excessive reactive oxygen species (ROS), together with impaired antioxidant defense systems, are considered the main contributors to myocardial injury, structural damage, and apoptosis [3,4]. Nicotinamide adenine dinucleotide (NAD^+^) is a vital cofactor involved in glycolysis, oxidative phosphorylation, and cellular redox reactions [5]. To maintain redox homeostasis, NAD^+^ serves as an essential cofactor for sirtuins (Sirts), a family of NAD^+^-dependent deacetylases involved in the regulation of oxidative stress and mitochondrial function [6,7]. Among the Sirt family, *Sirt3*, which possesses strong NAD^+^-dependent deacetylation activity, plays a pivotal role in antioxidant defense by deacetylating and activating mitochondrial antioxidant systems, including superoxide dismutase 2 (SOD2) and glutathione (GSH)-related pathways [8]. Sirt3 is predominantly localized in mitochondria, where its enzymatic activity is directly dependent on intracellular NAD^+^ availability. Upon activation, Sirt3 deacetylates and activates SOD2, resulting in enhanced mitochondrial ROS scavenging and maintenance of redox homeostasis [9,10,11]. In contrast, NAD^+^ depletion impairs Sirt3 activity, leading to increased SOD2 acetylation and diminished antioxidant defense, which promotes oxidative stress and mitochondrial dysfunction [11].

Previous studies have revealed that depletion of NAD^+^ and downregulation of Sirt3 contribute to mitochondrial dysfunction, oxidative stress, inflammation (e.g., TNF-α, IL-1β, NF-κB activation), and apoptosis, all of which are implicated in the development and progression of cardiovascular diseases [5,12,13,14]. Given the critical role of NAD^+^ in Sirt3 activation and redox regulation, increasing attention has been directed toward CD38 (cluster of differentiation 38), a major NAD^+^-consuming enzyme located on the cell surface [15]. CD38 hydrolyzes NAD^+^, leading to reduced intracellular NAD^+^ availability and limiting the activity of NAD^+^-dependent enzymes such as Sirt3 [11]. Consequently, increased CD38 expression can impair Sirt3-mediated antioxidant defense and promote oxidative stress under pathological conditions [11]. Previous studies have demonstrated that CD38 is implicated in multiple pathological conditions, including aging, obesity, and diabetes, cardiac hypertrophy, and inflammation, through depletion of NAD^+^ [12,16,17]. Moreover, elevated CD38 expression has been shown to be involved in tissue injury, such as kidney and heart tissue injury, under pathological conditions, including cisplatin-induced toxicity [15,18,19,20]. Because CD38-mediated NAD+ depletion impairs Sirt3 activity and disrupts antioxidant defense systems, CD38-Sirt3 has emerged as a potential regulator of oxidative stress-related cardiac injury; however, cisplatin-induced cardiotoxicity through CD38 has not yet been investigated in rats.

Apigenin, a natural flavonoid compound, has gained commercial interest in the food and flavor industries due to its various biological activities, particularly its antioxidant and anti-inflammatory, antiapoptotic, and antitumor effects [21,22]. Consistent with these beneficial properties, growing evidence has demonstrated that apigenin can protect against various cardiovascular-related disorders, including myocardial ischemic injury, chronic metabolic disorders, and doxorubicin-induced cardiotoxicity [23,24,25]. Interestingly, previous studies have reported that apigenin suppresses not only oxidative stress-related diseases but also the growth of various types of cancer cell [26,27,28]. As oxidative stress is one of the major mechanisms involved in various pathological conditions, the protective effects of apigenin have been partly attributed to its ability to inhibit CD38 both in vitro and in vivo models [29,30]. However, potential involvement of CD38-Sirt3 signaling in the cardioprotective effects of apigenin in cisplatin-induced cardiotoxicity have not yet been investigated in rats. Therefore, this study aimed to investigate these protective effects, focusing on apigenin’s underlying mechanisms in rat hearts, which may involve oxidative stress, inflammation, and apoptotic signaling pathways.

## 2. Results

### 2.1. Apigenin Protected LV Function in Rats with Cisplatin-Induced Cardiotoxicity

**Figure 1:**
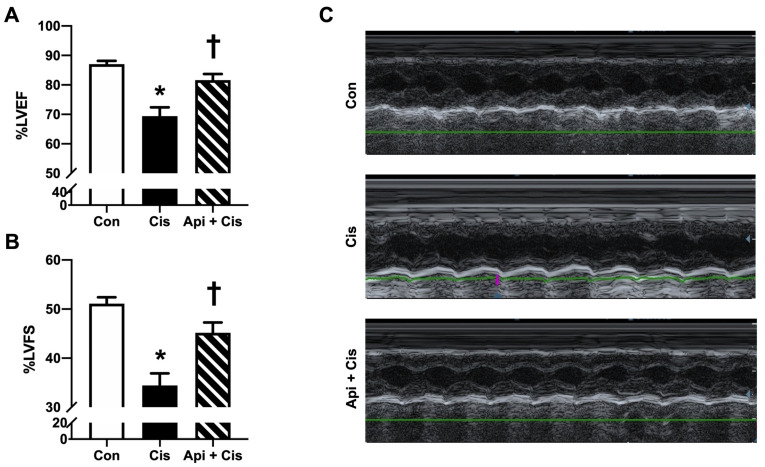
Effects of cisplatin (5 mg/kg) and apigenin pretreatment (50 mg/kg) on LV function in cisplatin-induced cardiotoxicity. (**A**) %LVEF, *n* = 8 per group; (**B**) %LVFS, *n* = 8 per group; and (**C**) M-mode echocardiographic images. * *p* < 0.05 vs. Con and † *p* < 0.05 vs. Cis (one-way ANOVA followed by Tukey’s post hoc test). Data were expressed as mean ± S.E.M. Con: rats treated with vehicle; Cis: rats treated with cisplatin at 5 mg/kg; Api + Cis: rats pretreated with apigenin at 50 mg/kg plus cisplatin 5 mg/kg; LVEF: left ventricular ejection fraction; and LVFS: left ventricular fractional shortening.

Rats treated with cisplatin exhibited impaired LV function, as evidenced by decreases in %LV ejection fraction and fractional shortening (%LVEF and %LVFS) in the cisplatin-treated group compared with the control group (Figure 1A,B). This demonstrated that a single dose of cisplatin administration at 5 mg/kg sufficiently impaired LV function in rats. However, pretreatment with apigenin at 50 mg/kg significantly increased %LVEF and %LVFS toward normal levels in the apigenin plus cisplatin-treated group, when compared to the cisplatin-treated group (Figure 1A,B). These findings suggest that pretreatment with apigenin at 50 mg/kg for 8 consecutive days exerted cardioprotective effects by preserving LV function in cisplatin-induced cardiotoxic rats. Representative echocardiographic images of the control, cisplatin-treated, and apigenin plus cisplatin-treated groups are shown in Figure 1C.

### 2.2. Apigenin Attenuated Cardiac Injury and Oxidative Stress in Rats with Cisplatin-Induced Cardiotoxicity

**Figure 2:**
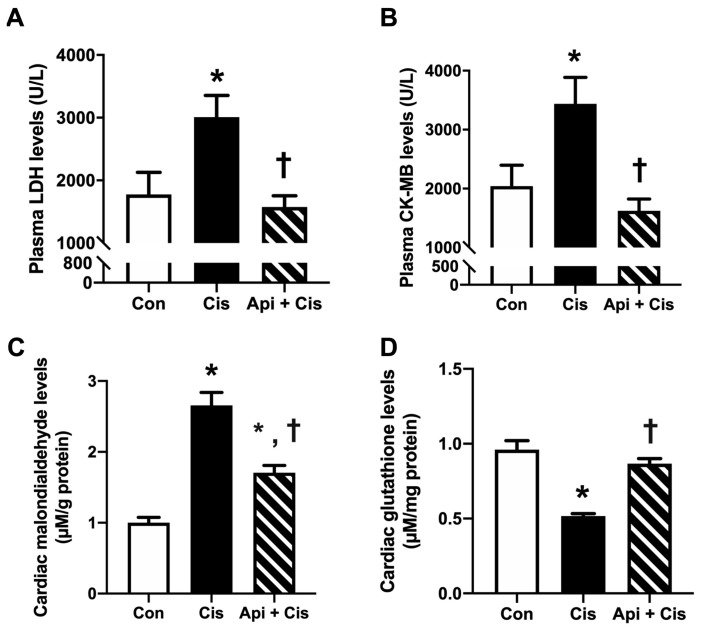
Effects of cisplatin (5 mg/kg) and apigenin pretreatment (50 mg/kg) on cardiac injury and cardiac oxidative stress status in cisplatin-induced cardiotoxicity. (**A**) plasma LDH levels, *n* = 8 per group; (**B**) plasma CK-MB levels, *n* = 8 per group; (**C**) cardiac MDA levels, *n* = 8 per group; and (**D**) cardiac GSH levels, *n* = 8 per group. * *p* < 0.05 vs. Con and † *p* < 0.05 vs. Cis (one-way ANOVA followed by Tukey’s post hoc test). Data were expressed as mean ± S.E.M. Con: rats treated with vehicle; Cis: rats treated with cisplatin at 5 mg/kg; Api + Cis: rats pretreated with apigenin at 50 mg/kg plus cisplatin 5 mg/kg; LDH: lactate dehydrogenase; CK-MB: creatine kinase-myocardial band; MDA: malondialdehyde; GSH: glutathione.

Cardiac injury was observed in the cisplatin-treated group, as indicated by the significantly elevated plasma lactate dehydrogenase (LDH) and creatine kinase-myocardial band (CK-MB) levels (Figure 2A,B). In addition, oxidative stress markers associated with cardiac injury, including malondialdehyde (MDA) and GSH, were further investigated in this study. The results showed that cardiac MDA levels significantly increased in the cisplatin-treated group compared with the control group (Figure 2C,D). In contrast, cardiac GSH levels were significantly reduced in the cisplatin-treated group compared with the control group (Figure 2C,D). These results suggest that cisplatin administration at 5 mg/kg successfully induced cardiac injury and oxidative stress in rats.

Pretreatment with apigenin at 50 mg/kg for 8 consecutive days significantly reduced plasma LDH and CK-MB levels in cisplatin-treated rats toward normal levels compared with the control cisplatin-treated group (Figure 2A,B). Similarly, apigenin at 50 mg/kg also significantly reduced cardiac MDA levels in cisplatin-treated rats, while increasing cardiac GSH back to normal levels (Figure 2C,D). These findings suggest that pretreatment with apigenin at 50 mg/kg for 8 consecutive days demonstrated cardioprotective effects by not only reducing cardiac injury but also attenuating cardiac oxidative stress and restoring antioxidant balance in cisplatin-induced cardiotoxic rats.

### 2.3. Apigenin Was Associated with Modulation of Cardiac CD38-Sirt3 Antioxidant Signaling Pathway in Rats with Cisplatin-Induced Cardiotoxicity

**Figure 3:**
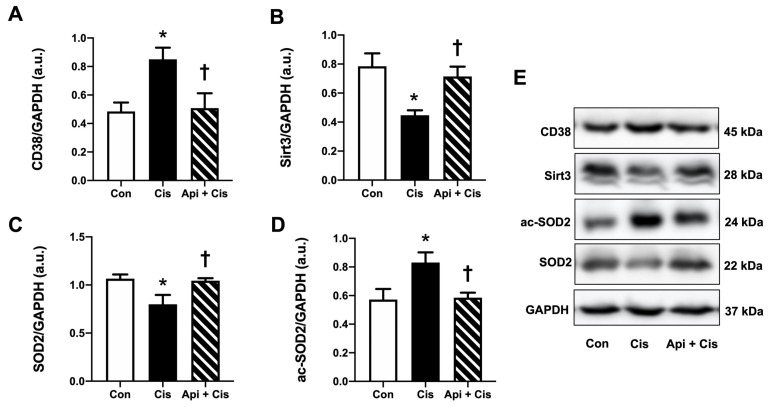
Effects of cisplatin (5 mg/kg) and apigenin pretreatment (50 mg/kg) on cardiac oxidative stress-related proteins in cisplatin-induced cardiotoxicity. (**A**) cardiac CD38 levels, *n* = 8 per group; (**B**) cardiac Sirt3 levels, *n* = 8 per group; (**C**) cardiac SOD2 levels, *n* = 8 per group; (**D**) cardiac ac-SOD2 levels, *n* = 8 per group; and (**E**) representative Western blots. GAPDH was used as a loading control. * *p* < 0.05 vs. Con and † *p* < 0.05 vs. Cis (one-way ANOVA followed by Tukey’s post hoc test). Data were expressed as mean ± S.E.M. Con: rats treated with vehicle; Cis: rats treated with cisplatin at 5 mg/kg; Api + Cis: rats pretreated with apigenin at 50 mg/kg plus cisplatin 5 mg/kg; CD38: cluster of differentiation 38; Sirt3: sirtuin 3; SOD2: superoxide dismutase 2; ac-SOD2: acetylated superoxide dismutase 2 at lysine 68; and GAPDH: glyceraldehyde 3-phosphate dehydrogenase.

In addition to observing the cardiac oxidative stress in cisplatin-treated rats, the expression of cardiac CD38, a major NAD^+^-consuming enzyme implicated in the regulation of cellular redox balance, was also investigated. The results demonstrate that cardiac CD38 expression was significantly increased in rats in the cisplatin-treated group compared with the control group (Figure 3A). Consistently with cardiac CD38 expression, cardiac Sirt3 was significantly decreased in rats in the cisplatin-treated group compared with the control group (Figure 3B). In addition, cardiac SOD2 and acetylated SOD2 at lysine 68 (ac-SOD2) levels were also quantified to further assess alterations in cardiac antioxidant defense in cisplatin-treated rats. Consistent with the above findings, cardiac SOD2 levels were significantly reduced in cisplatin-treated rats compared with the control group, whereas ac-SOD2 levels were significantly increased (Figure 3C,D). These findings indicated that cisplatin at 5 mg/kg impaired cardiac antioxidant defense system and induced cardiac oxidative stress, with a potential involvement of CD38-Sirt3 signaling pathway in rats.

Pretreatment with apigenin at 50 mg/kg for 8 consecutive days significantly reduced cardiac CD38 expression while significantly increasing cardiac Sirt3 and SOD2 expression levels in cisplatin-treated rats compared with the cisplatin-treated group (Figure 3A–C). Additionally, cardiac ac-SOD2 expression levels were significantly decreased toward normal levels in cisplatin-treated rats when compared with the cisplatin-treated group (Figure 3D). Representative Western blot images are shown in Figure 3E. These findings suggest that pretreatment with apigenin at 50 mg/kg was associated with modulation of CD38-Sirt3 signaling-related proteins and promoted antioxidant defense in cisplatin-induced cardiotoxic rats.

### 2.4. Apigenin Reduced Cardiac Inflammation in Rats with Cisplatin-Induced Cardiotoxicity

**Figure 4:**
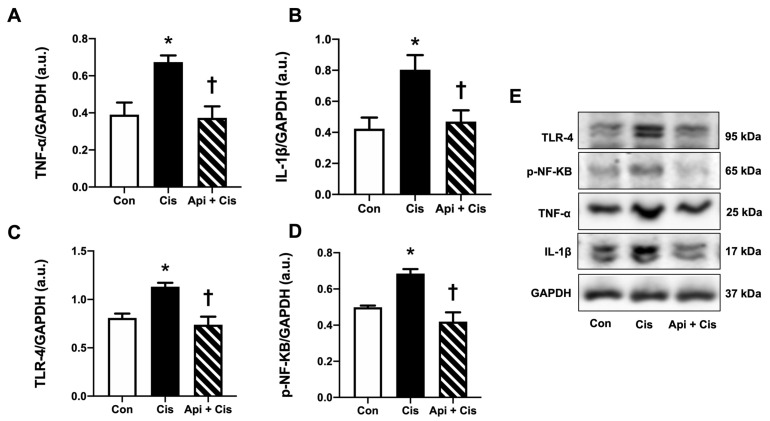
Effects of cisplatin (5 mg/kg) and apigenin pretreatment (50 mg/kg) on cardiac inflammatory responses in cisplatin-induced cardiotoxicity. (**A**) cardiac TNF-α levels, *n* = 8 per group; (**B**) cardiac IL-1β levels, *n* = 8 per group; (**C**) cardiac TLR-4 levels, *n* = 8 per group; (**D**) cardiac p-NF-κB levels, *n* = 8 per group; and (**E**) representative Western blots. GAPDH was used as a loading control. * *p* < 0.05 vs. Con and † *p* < 0.05 vs. Cis (one-way ANOVA followed by Tukey’s post hoc test). Data were expressed as mean ± S.E.M. Con: rats treated with vehicle; Cis: rats treated with cisplatin at 5 mg/kg; Api + Cis: rats pretreated with apigenin at 50 mg/kg plus cisplatin 5 mg/kg; TNF-α: tumor necrosis factor alpha; IL-1β: interleukin-1 beta; TLR-4: toll-like receptor 4; p-NF-κB: phosphorylation of nuclear factor kappa-light-chain-enhancer of activated B cells; and GAPDH: glyceraldehyde 3-phosphate dehydrogenase.

Administration of cisplatin at 5 mg/kg significantly elevated cardiac protein expression of tumor necrosis factor alpha (TNF-α) and interleukin-1 beta (IL-1β) levels in cisplatin-treated rats compared with the control group (Figure 4A,B). Since inflammatory cytokine expression was increased in cisplatin-treated rats, this study further investigated the expression of toll-like receptor 4 (TLR-4), a potent inflammatory receptor. The results showed that cardiac TLR-4 protein expression was significantly increased in cisplatin-treated rats compared with the control group (Figure 4C). Similarly, the cardiac phosphorylation of nuclear factor kappa-light-chain-enhancer of activated B cells (p-NF-κB), an upregulator of pro-inflammatory cytokines, was increased in cisplatin-treated rats compared with the control group (Figure 4D). This suggests that cisplatin at 5 mg/kg induced cardiac inflammation in rats.

Pretreatment with apigenin at 50 mg/kg for 8 consecutive days alleviated cardiac inflammation. This was indicated by significantly reduced expression levels of inflammatory markers, including TNF-α, TLR-4, p-NF-κB, and IL-1β, in apigenin-treated rats with cisplatin compared with the cisplatin-treated group (Figure 4A–D). Representative Western blot images are shown in Figure 4E. These findings suggest that pretreatment with apigenin at 50 mg/kg effectively reduced cardiac inflammation in cisplatin-induced cardiotoxic rats.

**Figure 5:**
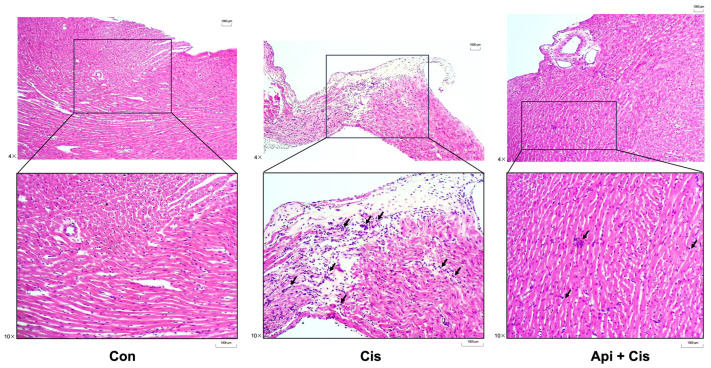
Representative images of cardiac tissue stained with H&E showing the effects of cisplatin at 5 mg/kg and pretreatment with apigenin at 50 mg/kg on histopathological changes at 100× magnification. Black arrows indicate inflammatory cell infiltration in cardiac tissue. Con: rats treated with vehicle; Cis: rats treated with cisplatin at 5 mg/kg; Api + Cis: rats pretreated with apigenin at 50 mg/kg plus cisplatin 5 mg/kg; and H&E: hematoxylin and eosin.

Representative cardiac sections were stained with hematoxylin and eosin (H&E) to assess histopathological alterations in the heart. As shown in Figure 5, the control group showed normal cardiac structure with regular arrangement of cardiac fibers. In contrast, the cisplatin-treated group displayed inflammatory cell infiltration (black arrows) and disruption of cardiac organization. Representative myocardial sections in the apigenin plus cisplatin group showed reduced inflammatory cell infiltration and improved preservation of cardiac structure compared with the cisplatin-treated group.

### 2.5. Apigenin Alleviated Cardiac Apoptosis in Rats with Cisplatin-Induced Cardiotoxicity

**Figure 6:**
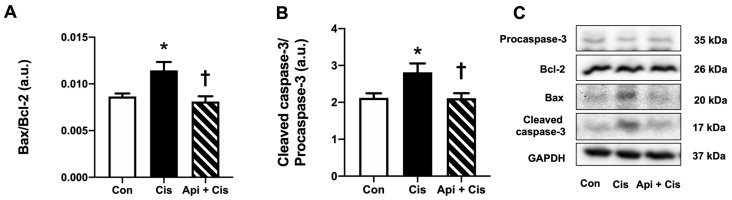
Effects of cisplatin (5 mg/kg) and apigenin pretreatment (50 mg/kg) on cardiac apoptosis in cisplatin-induced cardiotoxicty. (**A**) cardiac Bax/Bcl-2 levels, *n* = 8 per group; (**B**) cardiac cleaved caspase-3/procaspase-3 levels, *n* = 8 per group; and (**C**) representative Western blots. GAPDH was used as a loading control. * *p* < 0.05 vs. Con and † *p* < 0.05 vs. Cis (one-way ANOVA followed by Tukey’s post hoc test). Data were expressed as mean ± S.E.M. Con: rats treated with vehicle; Cis: rats treated with cisplatin at 5 mg/kg; Api + Cis: rats pretreated with apigenin at 50 mg/kg plus cisplatin 5 mg/kg; Bax: Bcl-2-associated X; Bcl-2: B-cell lymphoma 2; and GAPDH: glyceraldehyde 3-phosphate dehydrogenase.

Since cardiac injury and inflammation were induced by cisplatin administration, cardiac cell death (apoptosis) was observed in this study. The results show that the expression levels of cardiac apoptosis markers, including the Bcl-2-associated X to B-cell lymphoma 2 ratio (Bax/Bcl-2) and cleaved caspase-3/procaspase-3, were significantly elevated in cisplatin-treated rats compared with the control group (Figure 6A,B). This suggested that cisplatin at 5 mg/kg induced cardiac apoptosis in rats. In contrast, pretreatment with apigenin at 50 mg/kg led to a significant reduction in cardiac Bax/Bcl-2 and cleaved caspase-3/procaspase-3 expression compared with the cisplatin-treated group (Figure 6A,B). Representative Western blot images are shown in Figure 6C. The results suggest that pretreatment with apigenin at 50 mg/kg had a protective effect by reducing cardiac apoptosis in cisplatin-induced cardiotoxic rats.

## 3. Discussion

**Figure 7:**
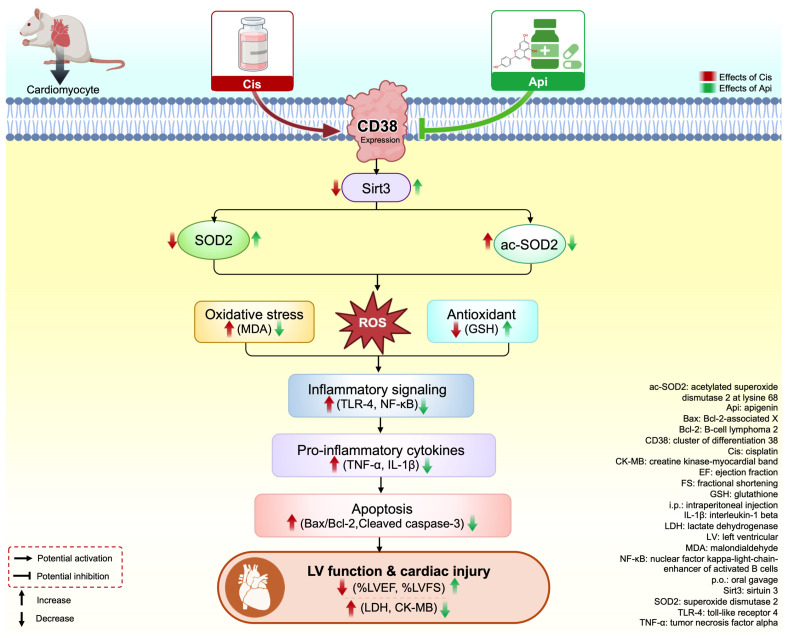
Proposed mechanism underlying the cardioprotective effects of apigenin against cisplatin-induced cardiotoxicity in rats. ac-SOD2: acetylated superoxide dismutase 2 at lysine 68; Api: apigenin; Bax: Bcl-2-associated X; Bcl-2: B-cell lymphoma 2; CD38: cluster of differentiation 38; Cis: cisplatin; CK-MB: creatine kinase-myocardial band; EF: ejection fraction; FS: fractional shortening; GSH: glutathione; i.p.: intraperitoneal injection; IL-1β: interleukin-1 beta; LDH: lactate dehydrogenase; LV: left ventricular; MDA: malondialdehyde; NF-κB: nuclear factor kappa-light-chain-enhancer of activated B cells; p.o.: oral gavage; Sirt3: sirtuin 3; SOD2: superoxide dismutase 2; TLR-4: toll-like receptor 4; TNF-α: tumor necrosis factor alpha.

There are several main findings from this study: (1) Administration of cisplatin at 5 mg/kg exhibited LV dysfunction, as evidenced by decreased %LVEF and %LVFS in cisplatin-treated rats. This functional impairment was associated with upregulated cardiac CD38 expression, which may contribute to oxidative stress and inflammatory responses, leading to cardiac injury and cardiac apoptosis in these rats. (2) Pretreatment with apigenin at 50 mg/kg for 8 consecutive days protected against cardiac dysfunction in rats with cisplatin-induced cardiotoxicity by alleviating cardiac injury, oxidative stress, inflammation, and apoptosis. To our knowledge, this is the first study to demonstrate that these cardioprotective effects are associated with suppression of cardiac CD38 expression and modulation of the Sirt3 signaling pathway in rats with cisplatin-induced cardiotoxicity. A summary of the cardioprotective effects of apigenin in cisplatin-induced cardiotoxicity in rats is shown in Figure 7.

Cisplatin has been widely used as a standard treatment for various cancers; however, its clinical use is restricted due to its dose-dependent toxic effects [31]. Growing evidence suggests that oxidative stress plays a crucial role in the development of cisplatin-induced cardiotoxicity [2,32,33]. One of the characteristic manifestations of cisplatin-induced cardiotoxicity is LV dysfunction [33]. In the present study, cisplatin-treated rats exhibited decreased %LVEF and %LVFS, along with elevated plasma LDH and CK-MB levels, indicating the presence of LV dysfunction and injury. Collectively, these findings support the notion that cisplatin at 5 mg/kg impairs LV function and promotes cardiac damage. At the molecular level, disruption of NAD^+^ homeostasis has been implicated in the progression of cisplatin-induced toxicities through elevated ROS production [34]. CD38 is a major NAD^+^-consuming enzyme that regulates intracellular NAD^+^ levels and plays a critical role in cellular redox homeostasis [15]. To our knowledge, the present study is the first to demonstrate that cisplatin remarkedly increased cardiac CD38 expression, which was associated with increased cardiac oxidative stress, as evidenced by elevated cardiac MDA levels and decreased cardiac GSH levels in rats. Given the essential role of NAD^+^ in antioxidant defense mechanisms [35], our findings suggest that CD38 upregulation may contribute to cisplatin-induced oxidative stress. Furthermore, Sirt3, a downstream NAD^+^-dependent deacetylase associated with the CD38-NAD^+^ signaling pathway [36], was significantly decreased in cisplatin-treated rats. This reduction was accompanied by increased cardiac ac-SOD2 at lysine 68 expression, indicating decreased Sirt3-mediated deacetylation of SOD2. Since Sirt3-mediated deacetylation enhances SOD2 antioxidant activity [37], the increased cardiac ac-SOD2 expression together with decreased cardiac SOD2 expression may implicate impaired SOD2 antioxidant defense in cisplatin-treated rats. Taken together, the observed alterations in CD38-Sirt3 signaling and SOD2 may plausibly contribute to excessive ROS accumulation and oxidative stress, leading to impaired cardiac function and cardiac injury in cisplatin-treated rats. Our data provide evidence suggesting alterations in CD38-Sirt3 signaling; however, further studies are required to investigate the NAD^+^ levels, as well as CD38 and Sirt3 activities, in a cisplatin-induced cardiotoxicity model.

In addition, there are several reports that oxidative stress can further trigger inflammatory response and apoptosis following cisplatin administration [31,38,39]. Consistent with these observations, increased cardiac TNF-α, IL-1β, and p-NF-κB expression, together with elevated Bax/Bcl-2 and cleaved caspase-3/procaspase-3 ratios, were observed in cisplatin-treated rats in the present study. Moreover, TLR-4 is major pattern recognition receptor of the innate immune system that recognizes lipopolysaccharide (LPS) and activates downstream inflammatory signaling pathways [40,41]. In addition to LPS, a previous study has demonstrated that cisplatin can directly activate TLR-4 signaling [42]. Therefore, the increased cardiac TLR-4 expression observed in this study can contribute to cardiac NF-κB activation and the subsequent production of cardiac pro-inflammatory cytokines, including TNF-α and IL-1β, leading to cardiac inflammation and injury in cisplatin-treated rats.

Apigenin is one of the most powerful and widely studied flavonoids, exhibiting multiple beneficial biological activities with low toxicity [21,43]. Accumulating evidence indicates that flavonoids as a class exert cardioprotective effects in various models of cardiovascular injury. Representative flavonoids, including apigenin, rutin, and isorhamnetin, have been reported to attenuate cardiac damage through their antioxidant, anti-inflammatory, and anti-apoptotic properties [22,44,45,46]. Recently, apigenin has attracted increasing attention in supportive cancer care research because of its potent antioxidant, anti-inflammatory, and anticancer properties [26,27,28,47]. Consistent with these biological activities, the present study demonstrated that pretreatment with apigenin attenuated cisplatin-induced LV dysfunction, as evidenced by improved %LVEF and %LVFS, along with normalization of cardiac injury biomarkers, including LDH and CK-MB levels in cisplatin-treated rats. It is well documented that apigenin effectively attenuates oxidative stress and enhances endogenous antioxidant defenses through direct free radical scavenging activity attributable to its flavonoid structure in various disease models, including doxorubicin-induced cardiotoxicity [21,47,48]. Consistent with these reports, pretreatment with apigenin significantly reduced cardiac MDA levels and restored cardiac GSH levels in cisplatin-treated rats. As discussed above, CD38 plays a role in the regulation of oxidative stress. Recently, apigenin has been identified as a direct CD38 inhibitor, leading to increased intracellular NAD+ and activation of the NAD^+^-dependent signaling pathway [29]. In the present study, pretreatment with apigenin reduced cardiac CD38 expression while increasing cardiac Sirt3 and SOD2 expression levels in cisplatin-treated rats. In addition, pretreatment with apigenin decreased cardiac ac-SOD2 expression, suggesting enhanced Sirt3-mediated deacetylation of SOD2. Although direct inhibition of cardiac CD38 activity was not observed in the present study, our findings provide evidence suggesting a potential involvement of CD38-Sirt3 signaling in the antioxidant and cardioprotective effects of apigenin against cisplatin-induced cardiotoxicity.

Pretreatment with apigenin alleviated cardiac oxidative stress in cisplatin-induced cardiotoxicity and further suppressed inflammatory cytokines, as indicated by reduced cardiac TNF-α, IL-1β expression. Furthermore, previous studies have found that apigenin suppressed pro-inflammatory cytokine production through inhibiting TLR-4 and inactivation of the NF-κB signaling pathway [49,50]. Consistent with these findings, the present study demonstrated that apigenin decreased cardiac TLR-4 and cardiac p-NF-κB expression, suggesting that this effect contributes to the suppression of inflammatory cytokine production in cisplatin-treated rats. Although p-NF-κB was assessed as an indicator of NF-κB pathway activation, evaluation of total NF-κB expression may provide a more comprehensive understanding of the inflammatory pathway. This study also showed that pretreatment with apigenin reduced cisplatin-induced cardiac apoptosis in rats, as indicated by decreased cardiac Bax/Bcl-2 and cleaved caspase-3/procaspase-3 ratios in rats. This may be because both oxidative stress and inflammation are closely associated with the activation of apoptotic pathways in cardiomyocytes following cisplatin exposure [44]. Taken together, our data demonstrate that apigenin exerts significant cardioprotective effects against cisplatin-induced cardiotoxicity by improving LV function and against cardiac injury by suppressing oxidative stress, inflammation, and apoptosis, with potential involvement of CD38-Sirt3 signaling.

This study has some limitations. Although histopathological assessment was qualitative, representative images showed distinct differences among groups. Future quantitative histopathological analyses may further strengthen these findings. Furthermore, although apigenin has been reported to exhibit low toxicity, an apigenin-alone group was not included in the present study; the independent effects of apigenin warrant further investigation. Nevertheless, the present findings provide supportive evidence that apigenin attenuates cisplatin-induced cardiotoxicity and suggest a potential involvement of CD38-Sirt3 signaling in its cardioprotective effects. The present findings further support a potential association between CD38-Sirt3 signaling alterations and cisplatin-induced cardiotoxicity, as well as the observed effects of apigenin, consistent with an associative interpretation of the data. Together, these findings provide initial insight into the potential role of CD38-Sirt3 signaling in cisplatin-induced cardiotoxicity and the cardioprotective effects of apigenin, serving as a foundation for future mechanistic investigations.

## 4. Materials and Methods

### 4.1. Experimental Animals

A total of twenty-four male Sprague Dawley rats weighing 200–220 g were purchased from Nomura Siam International (M-CLEA Bioresource Co., ltd., Bangkok, Thailand). All rats were housed in a temperature-controlled room (22–24 °C) under a 12 h dark/light cycle, with ad libitum access to standard chow diet and water. After a 7-day acclimatization period, rats were randomly assigned to three groups (*n* = 8 per group): (1) control group, (2) cisplatin group, and (3) apigenin plus cisplatin group.

Rats in the control (Con) group received normal saline solution (NSS) via daily oral gavage (p.o.) for 5 days, followed by a single intraperitoneal injection (i.p.) of NSS as the vehicle, with continued NSS administration for an additional 3 days. Rats in the cisplatin (Cis) group received NSS via daily p.o. for 5 days prior to a single dose of cisplatin (5 mg/kg, i.p.), followed by continued vehicle treatment for an additional 3 days. Rats in apigenin plus cisplatin (Api + Cis) group were treated with apigenin (purity > 99%; 75 mg/capsule; Lot No. N401AN; Renue by Science, Jacksonville, FL, USA), which was freshly suspended in distilled water and vortexed thoroughly prior to oral administration, at a dose of 50 mg/kg/day/p.o. [51,52] for 5 days prior to a single dose of cisplatin (5 mg/kg, i.p.). Apigenin treatment was then continuously administered for an additional 3 days following cisplatin injection.

**Figure 8:**
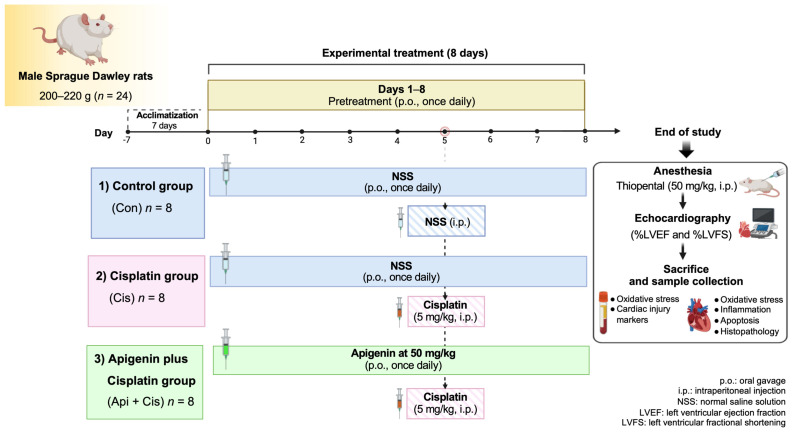
Schematic illustration of the experimental protocol. p.o.: oral gavage; i.p.: intraperitoneal injection; NSS: normal saline solution; LVEF: left ventricular ejection fraction; and LVFS: left ventricular fractional shortening.

At the end of the study protocol, rats were anesthetized with thiopental (50 mg/kg, i.p.) to determine LV performance using echocardiography. Then, the rats were sacrificed and blood from the abdominal aorta was collected to examine cardiac injury and systemic oxidative stress markers. The heart was rapidly excised for molecular analysis, including analyses of cardiac oxidative stress, cardiac inflammation, and cardiac apoptosis and histopathological examination of cardiac structure. The experimental protocol is illustrated in Figure 8.

### 4.2. Assessment of LV Function

Rats’ LV function was assessed using echocardiography (Mindray DC-70 Ultrasound System, Shenzhen, China) under light anesthesia. The papillary muscle level was identified, and M-mode echocardiographic images were obtained to evaluate LV ejection fraction %LVEF and %LVFS. All parameters were calculated as the average of three consecutive cardiac cycles, with the investigator blinded to the experimental groups.

### 4.3. Assessment of Cardiac Injury

Plasma LDH and CK-MB levels were used to indicate myocardial damage [53,54]. Plasma LDH levels were measured using an LDH toxicity assay (Roche Cobas, Roche, Basel, Switzerland) with an automated biochemical analyzer. Briefly, the plasma LDH catalyzes the conversion of L-lactate to pyruvate, leading to NADH production. The rate of production is proportional to LDH enzymatic activity, and increased absorbance reflects increased plasma LDH levels.

The CK-MB levels were measured within 24 h after blood collection using the CK-MB isoenzyme assay with an automatic biochemical analyzer (Roche Cobas, Roche, Basel, Switzerland), according to the manufacturer’s instructions.

### 4.4. Assessment of Cardiac Oxidative Stress

MDA, a lipid peroxidation byproduct, was used as an indicator of oxidative stress in cardiac tissue. Briefly, MDA reacts with thiobarbituric acid (TBA) at 90–100 °C to produce a pink-colored thiobarbituric acid reactive substance (TBARS). Cardiac TBARS levels were measured using the TBARS assay kit (Cayman Chemical, Ann Arbor, MI, USA) according to the manufacturer’s instructions. The levels of cardiac TBARS were calculated based on the standard curve and expressed as cardiac MDA levels.

GSH levels, an indicator of antioxidant capacity, were determined in cardiac tissue using a QuantiChrom GSH Assay Kit (Bioassay Systems, Hayward, CA, USA) according to the manufacturer’s instructions.

### 4.5. Assessment of Cardiac Histopathology

H&E staining was conducted to assess cardiac structural alterations in cardiac tissue. The tissue was perfusion-fixed with 4% paraformaldehyde, followed by cardiac tissue processing and embedding prior to H&E staining. Cardiac histopathological images were captured with a microscope (Eclipse Ts2, Nikon, Tokyo, Japan). Approximately 3–5 tissue sections were analyzed per animal under blinded conditions. Representative photomicrographs were captured at low-power (4×) and high-power (10×) magnifications and were randomly selected under blinded conditions.

### 4.6. Western Blot Analysis

Protein lysates were extracted from cardiac tissues and separated on 10% sodium dodecyl sulfate-polyacrylamide gel electrophoresis. The separated proteins were subsequently transferred onto nitrocellulose membranes using a wet/tank blotting system (Bio-Rad, Hercules, CA, USA) with glycine/methanol transfer buffer. Membranes were blocked for 1 h with either 5% bovine serum albumin or 5% skimmed milk prepared in Tris-buffered saline containing Tween 20, followed by overnight incubation at 4 °C with the primary antibodies listed in Table 1. After washing, the membranes were incubated with appropriate secondary antibodies for 1 h. Protein bands were visualized using enhanced chemiluminescence detection reagents (Bio-Rad, USA). Images were captured using a ChemiPRO Imaging System XS (Cleaver Scientific Ltd., Rugby, UK), and band intensities were analyzed using ImageJ software, version 1.53a (National Institutes of Health, Bethesda, MD, USA).

### 4.7. Statistical Analysis

Statistical analyses were performed using GraphPad Prism 8 software (GraphPad Software, San Diego, CA, USA). All data are presented as mean ± standard error of the mean (S.E.M.). Comparisons among multiple groups were analyzed using one-way analysis of variance (ANOVA) followed by Tukey’s post hoc test. Statistical significance was defined as a *p*-value less than 0.05.

## 5. Conclusions

This study is the first to show that cisplatin at 5 mg/kg is associated with alterations in CD38-Sirt3 signaling, LV dysfunction, cardiac injury, oxidative stress, inflammation, and apoptosis. Pretreatment with apigenin at 50 mg/kg effectively attenuated these alterations, as evidenced by improvements in LV function and injury, suppression of oxidative stress and inflammatory responses, and inhibition of cardiac apoptosis. Beyond its reported antioxidant, anti-inflammatory, and anti-apoptotic properties, these cardioprotective effects are potentially associated with CD38-Sirt3 signaling modulation and restoration of redox homeostasis in cisplatin-induced cardiotoxicity. Collectively, these findings suggest a potential role of apigenin in mitigating cisplatin-induced cardiotoxicity and warrant further investigation for potential application in supportive cancer care settings.

## Figures and Tables

**Table 1:** Primary antibodies used in this study.

Antibody	Manufacturer	Host	Dilution
ac-SOD2	Abcam	Rabbit	1:1000
Bax	Cell signaling	Rabbit	1:1000
Bcl-2	Cell signaling	Rabbit	1:1000
CD38	Santa Cruz	Rabbit	1:1000
Cleaved caspase-3	Cell signaling	Rabbit	1:1000
GAPDH	Cell signaling	Mouse	1:2000
IL-1β	Merck	Rabbit	1:1000
Phosphorylation of NF-κB	Cell signaling	Rabbit	1:1000
Procaspase-3	Cell signaling	Rabbit	1:1000
Sirt3	Cell signaling	Rabbit	1:1000
SOD2	Cell signaling	Rabbit	1:1000
TLR-4	Abcam	Rabbit	1:1000
TNF-α	Abcam	Rabbit	1:1000

ac-SOD2: acetylated superoxide dismutase 2 at lysine 68; SOD2: superoxide dismutase 2; Bax: Bcl-2-associated X; Bcl-2: B-cell lymphoma 2; CD38: cluster of differentiation 38; GAPDH: glyceraldehyde 3-phosphate dehydrogenase; IL-1β: interleukin-1 beta; NF-κB: nuclear factor kappa-light-chain-enhancer of activated B cells; Sirt3: sirtuin 3; TLR-4: toll-like receptor 4; TNF-α: tumor necrosis factor alpha.

## Data Availability

The original contributions presented in this study are included in the article. Further inquiries can be directed to the corresponding authors.

## Abbreviations

The following abbreviations are used in this manuscript:

ac-SOD2	acetylated superoxide dismutase 2 at lysine 68
Api + Cis	rats pretreated with apigenin at 50 mg/kg plus cisplatin 5 mg/kg
Bax	Bcl-2-associated X
Bcl-2	B-cell lymphoma 2
CD38	cluster of differentiation 38
Cis	rats treated with cisplatin at 5 mg/kg
CK-MB	creatine kinase-myocardial band
Con	rats treated with vehicle
DNA	deoxyribonucleic acid
GSH	glutathione
H&E	hematoxylin and eosin
IL-1β	Interleukin-1 beta
LDH	lactate dehydrogenase
MDA	malondialdehyde
NAD^+^	nicotinamide adenine dinucleotide
NF-κB	nuclear factor kappa-light-chain-enhancer of activated B cells
ROS	reactive oxygen species
Sirt3	sirtuin 3
TLR-4	toll-like receptor 4
TNF-α	tumor necrosis factor alpha
